# Fully-automated synthesis of ^177^Lu labelled FAPI derivatives on the module modular lab-Eazy

**DOI:** 10.1186/s41181-021-00130-3

**Published:** 2021-04-16

**Authors:** Kurtulus Eryilmaz, Benan Kilbas

**Affiliations:** Moltek A. S. Gebze Organize Sanayi, 41400 Gebze, Kocaeli, Turkey

**Keywords:** Automated synthesis, FAPI-04, FAPI-46, ^177^Lutetium, Therapeutic

## Abstract

**Background:**

To the best of our knowledge, manually production of [^177^Lu]Lu-FAPI radiopharmaceutical derivatives has been only described in literature. In this work, a fully-automated [^177^Lu]Lu-FAPI synthesis has been well designed for the first time using commercially available synthesis module. In addition to the development of an automated system with disposable cassette, quality control (QC) and stability studies were comprehensively presented.

**Results:**

A fully automated synthesis of [^177^Lu]Lu-FAPI derivatives was achieved on the Modular Lab Eazy (ML Eazy) with high radiochemical yield ([^177^Lu]Lu-FAPI-04; 88% ± 3, [^177^Lu]Lu-FAPI-46; 86% ± 3). Chromatographic analysis indicated the formation of radiosynthesis with an absolute radiochemical purity (99%). Stability experiments clarified the durability of the products within 4 days. All obtained specifications are consistent to European Pharmacopoeia.

**Conclusion:**

A fully automated synthesis of [^177^Lu]Lu-FAPI radiopharmaceuticals was accomplished regarding quality control standards and quality assurance by using commercially available a modular approach namely ML Eazy with disposable customized cassette and template.

**Graphical abstract:**

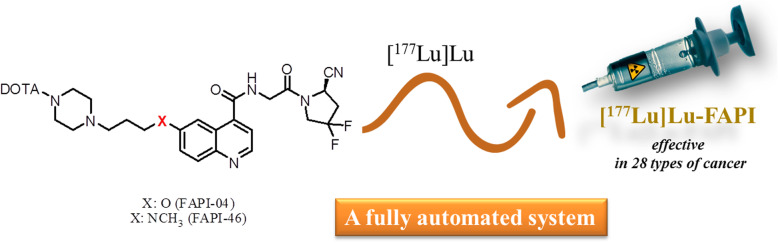

## Background

**Fig. 1:**
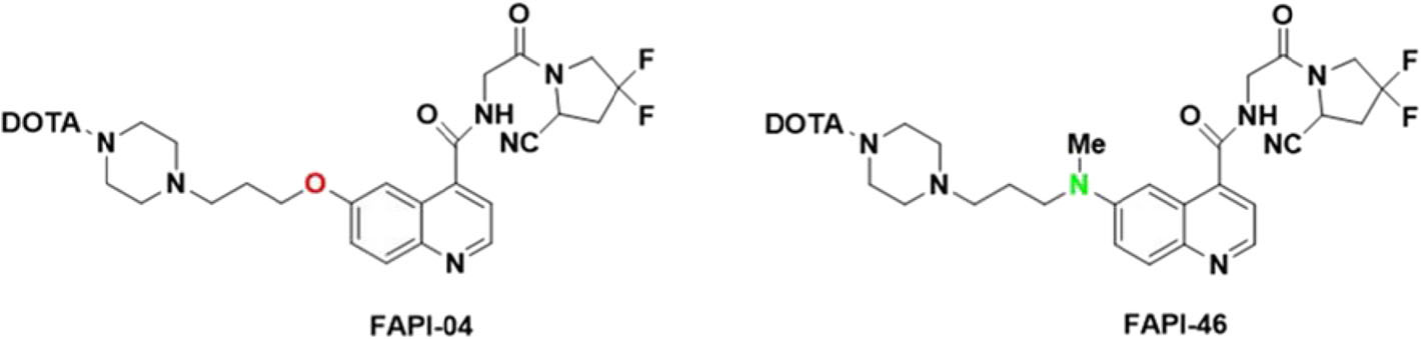
Molecular Structures of FAPI-04 and FAPI-46

**Fig. 2:**
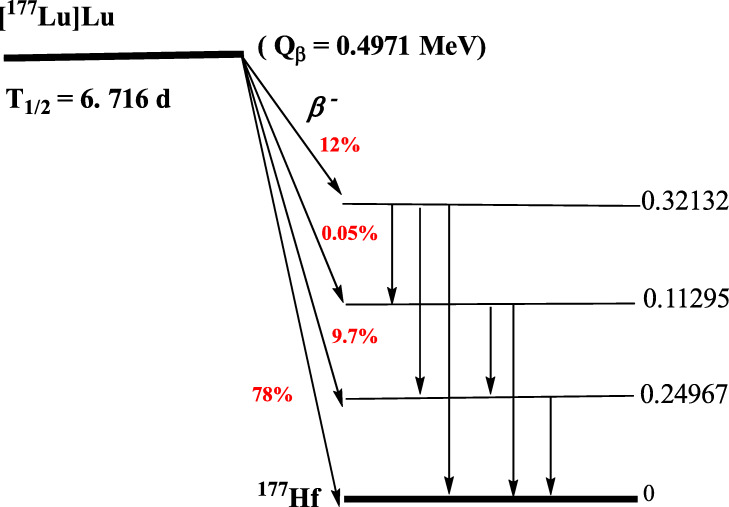
Decay chain scheme of [^177^Lu]Lutetium

Fibroblast activation protein (FAP, FAP-α), a type-II transmembrane serine protease acts on various hormones and extracellular matrix components which has an important role for tumor biology. (Kalluri 2016.). FAP is able to operate the tumor cell behavior, therefore it can be used as an imaging tracer for many cancer types particularly colorectal, ovarian, pancreatic, and hepatocellular carcinomas which are identified by a strong desmoplastic reaction (Gascard and Tlsty 2017; Siveke 2018). Recently, fibroblast activation protein-specific inhibitor (FAPI) decorated radiopharmaceuticals have been great of interest for the diagnosis of various tumor species (Fig. 1) (Lindner et al. 2018). For example, [^68^Ga]Ga-DOTA-FAPI-04 PET/CT exhibited excellent high-tumor uptake in clinically 28 different cancer types by contrast with low background in muscle and blood pool by fast imaging (Kratochwil et al. 2019). Those potentials such as specific target, high-tumor uptake with low background, rapid clearance from blood and fast diagnosis led to a new aspect for the development of theranostic studies based on FAPI derivatives (Ballal et al. 2020). Recently, FAPI precursor has been labelled by β-emitter radionuclides such as [^90^Y] Y and [^177^Lu] Lu in preclinical studies (Lindner et al. 2019). [^177^Lu] Lu, a β-emitter trivalent lanthanide type radionuclide has been frequently utilized for various palliative treatments such as lung cancer, prostate cancer, bone pain palliation etc. due to the ideal physical properties (*T*½ = 6.73 days, *E*β_max_ = 497 keV; *E*γ = 113, 208 keV) and it also provides image of tumour species by its γ-emitting property (Fig. 2) (Banerjee et al. 2015; Emmett et al. 2017). More recently, FAPI-46 was also successfully radiolabeled by ^225^[Ac] Ac and [^64^Cu] Cu radionuclides in preclinical study for the treatment of pancreatic cancer (Watabe et al. 2020). Those promising clinical and preclinical results provide preliminary evidence for the feasibility of theranostics of numerous malignant tumors using radiolabeled FAPI species.

Even exponential growth has been reported about applying FAPI based radiopharmaceuticals for various cancer treatments, those therapeutic studies have not been automatically performed yet. In this study, the aim was to describe a fully automated synthesis of [^177^Lu]Lu-FAPI radiopharmaceuticals regarding radiation safety and pharmaceutical requirements by using commercially available a modular approach namely ML Eazy. In addition to the description of an automated synthesis procedure, detailed stability and QC studies have been also exhibited.

## Methods

### Materials

[^177^Lu] Lu (n.c.a) was obtained from Isotopia Molecular Imaging Ltd., FAPI-04 (Purity ≥99%) was supplied from MedChemExpress LLC. and FAPI-46 (Purity ≥99%) compound was manufactured by ABX. Polatom’s lyophilized ascorbic acid buffer (50 mg Ascorbic acid + 7.9 mg NaOH, trace metal basis, and Sterile GMP product) was used and the disposable cassettes were from Eckert & Ziegler Eurotope GmbH. Purification cartridge CM (Sep-Pak Accell Plus CM Plus Light Cartridge, 130 mg Sorbent per Cartridge, 37–55 μm, WAT023531) was included in cassette accessories. Other chemicals and materials were purchased from Merck in ultra-pure and trace metal basis grade.

### Synthesis of [^177^Lu]Lu-FAPI-04 & [^177^Lu]Lu-FAPI-46

#### Preliminary studies

A series of manually radiolabeling studies have been performed to determine the optimum synthesis conditions of [^177^Lu]Lu-FAPI derivatives. In all experimental studies, medium of pH was 4.5 formed by ascorbic acid buffer system (lyophilized kit 50 mg Ascorbic acid + 7.9 mg NaOH dissolved in 1 mL ultrapure water), which provides the most suitable reaction condition for the complexation of [^177^Lu] Lu and DOTA chelator. The reaction temperature was kept constant at 95 °C. Radiochemical yield and radiochemical purity values were tried to be optimized by changing the peptide amount and the reaction time parameters. As a result of those studies, the optimum synthesis parameters, for FAPI-04 (Table 1, entry 4,5) and FAPI-46 (Table 2, entry 4,5) were determined as mCi / μg for the amount of peptide and 20 min at 95 °C for the reaction time.

**Table 1:** Preliminary studies of [^177^Lu]Lu-FAPI-04

	Amount of [^**177**^Lu] Lu (mCi)	Amount of Peptide (μg.)	Time (Min.)	Temp. (°C)	RCY%	RCP%
**1**	25	15	10	95	55	97.2
**2**	25	25	10	95	70	98.1
**3**	25	25	15	95	80	99.0
**4**	25	25	20	95	88	99.6
**5**	100	100	20	95	87	99.5

**Table 2:** Preliminary studies of [^177^Lu]Lu-FAPI-46

	Amount of [^**177**^Lu] Lu (mCi)	Amount of Peptide (μg.)	Time (Min.)	Temp. (°C)	RCY%	RCP%
**1**	25	15	10	95	50	97.0
**2**	25	25	10	95	66	97.7
**3**	25	25	15	95	77	98.8
**4**	25	25	20	95	85	99.4
**5**	100	100	20	95	86	99.6

*RCY*: Radiochemical yield, *RCP*: Radiochemical purity

**Table 3:** General Synthesis Steps

Step		Parameter
**1**	Pre-heating of reaction vial	80 °C (60 s.) (Fig. 3 c)
**2**	Transfer (Fig. 3 Red Line)	Transfer of [^177^Lu] Lu (Fig. 3 a) to the reaction vial by elution with radiolabelling solution (Fig. 3 b).
**3**	Radiolabelling	95 °C (20 min.) (Fig. 3 c)
**4**	Cooling & Dilution (Fig. 3 Yellow Line)	Transfer of saline (Fig. 3 d) to the reaction vial (Fig. 3 c)**.**
**5**	Transfer, Purification, Sterile Filtration (Fig. 3 Green Line)	Transfer of last product to the final vial (Fig. 3 e) by passing through Sep Pak CM cartridge and sterilization filter.

### Automated synthesis device and synthesis method

The optimum synthesis parameters for [^177^Lu]Lu-FAPI derivatives were determined by the data obtained from our preliminary studies which were directly transferred to the ML-Eazy synthesis device (Tables 1 and 2, entry 4,5). Moreover, the disposable cassettes pre-designed by Eckert & Ziegler for the synthesis of routine [^177^Lu]Lu-Peptides were used.

### Preparation of [^177^Lu]Lu-FAPI-04 & [^177^Lu]Lu-FAPI-46

**Fig. 3:**
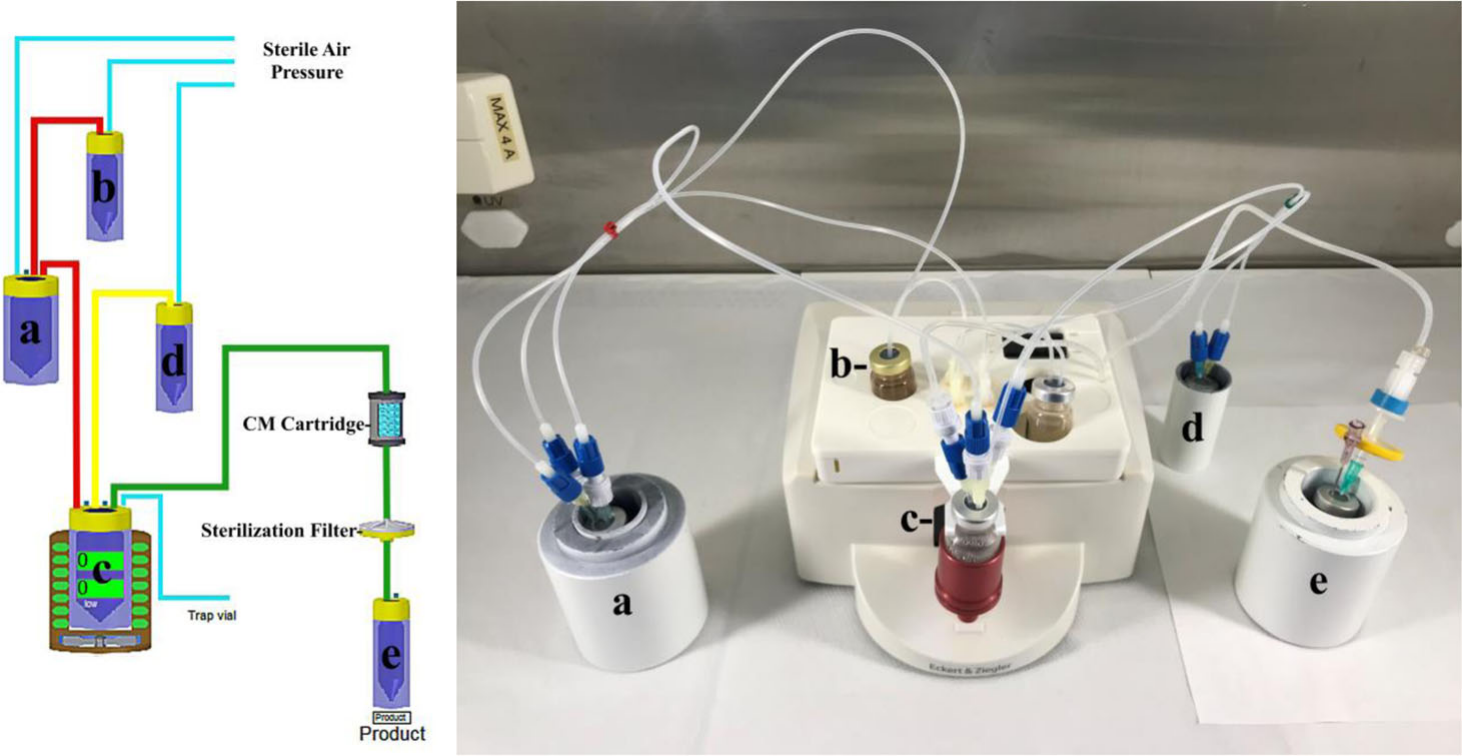
ML-Eazy fully automated synthesis device, disposable cassette and schematic flow (**a**) [^177^Lu] Lu vial, (**b**) Buffer and peptide vial, (**c**) Reaction vial and heater, (**d**) Saline vial, (**e**) Final product vial

Lyophilized ascorbic acid buffer (50 mg Ascorbic acid + 7.9 mg. NaOH) was dissolved in 1.0 mL of sterile ultrapure water (**pH 4.5**). After adding 100 μg /μL amount of FAPI-04 or FAPI-46, it was transferred to the vial (**b**) on the cassette (Fig. 3 b). 20 mL of saline was added to the saline vial and connected to its place on the cassette (Fig. 3 d). The CM cartridge was conditioned with 10 mL of sterile ultrapure water and connected to the final product transfer line along with the sterilization filter. Then, the final product vial (Fig. 3e) was connected to the end of the final product transfer line, the synthesis cassette was assembled to the synthesis device. Finally, 100 mCi [^177^Lu] Lu (in 100 μL, 0.04 M HCl) was connected to its place on the cassette (Fig. 3 a). After completion of synthesis, the final product [^177^Lu]Lu-FAPI-04 and [^177^Lu]Lu-FAPI-46 were obtained with 88% ± 3 and 86% ± 3 radiochemical yields respectively (Table 3).

### Characterization methods

HPLC analyzes were performed by combined Shimadzu LC20A and Eckert & Ziegler HPLC Scan devices, using ACE-3 C18 150 × 3.0 mm column. For TLC analyses ITLC-SG Agilent TLC plates and Eckert & Ziegler TLC Scan device were used.

### Quality controls of [^177^Lu]Lu-FAPI radiopharmaceuticals

**Fig. 4:**
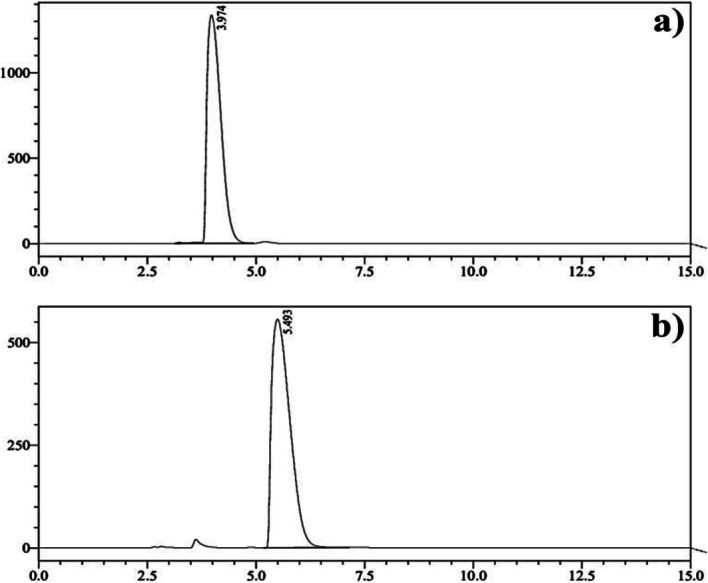
HPLC UV chromatograms of (**a**) FAPI-04 precursor (UV-254 nm), (**b**) FAPI-46 precursor (UV-264 nm) on a ACE-3 C18 150 × 3.0 mm column, mobile phase: %13 ACN / %87 Water (% 0,1 TFA), isocratic flow: 0.6 ml/min., FAPI-04 RT: 3.5–4.5 min., FAPI-46 RT: 5–6 min

**Fig. 5:**
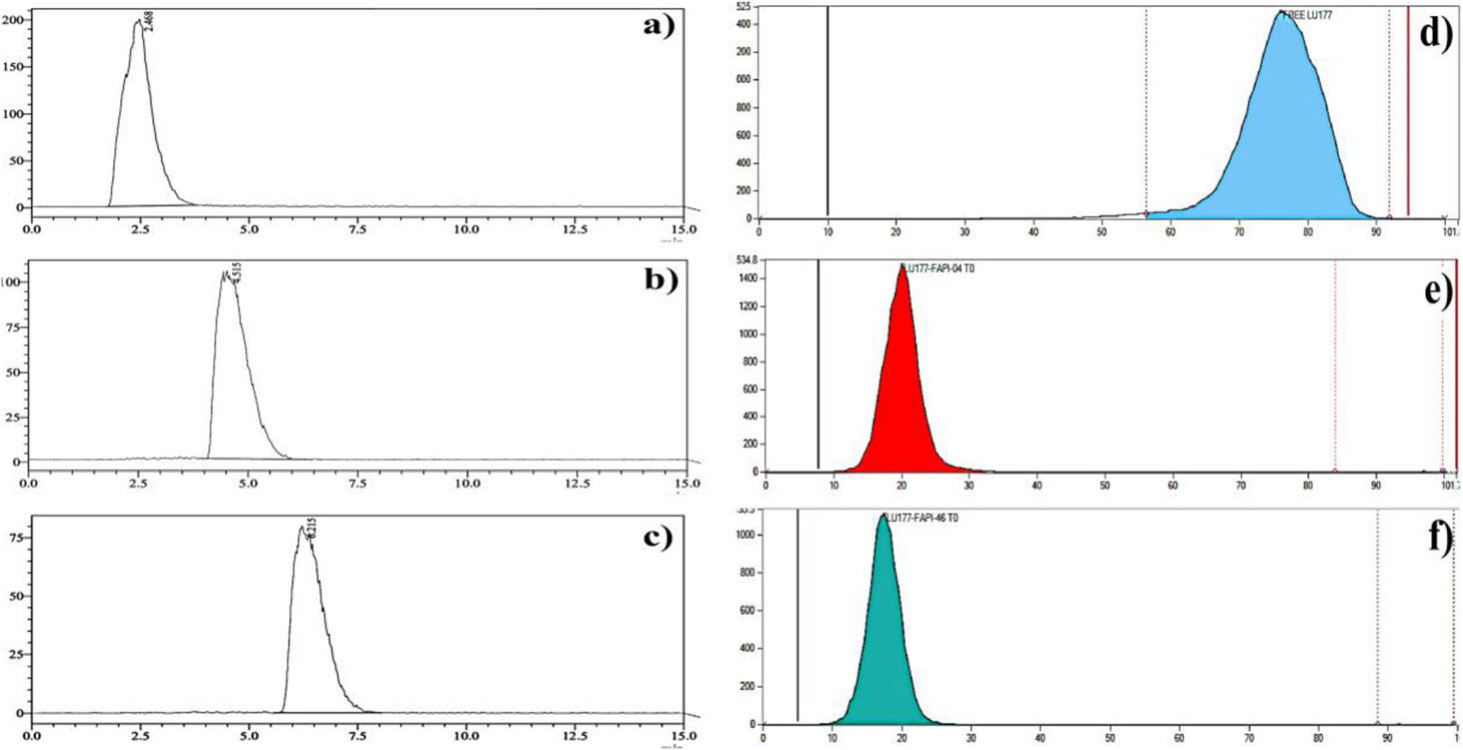
R-HPLC chromatograms of (**a**) Free [^177^Lu] Lu, (**b**) [^177^Lu]Lu-FAPI-04, (**c**) [^177^Lu]Lu-FAPI-46. Free [^177^Lu] Lu RT: 2–3 min., [^177^Lu]Lu-FAPI-04 RT: 4–5 min., [^177^Lu]Lu-FAPI-46 RT: 6–7 min. (Method parameters are the same as depicted in Fig. 2). R-TLC chromatograms of (**d**) Free [^177^Lu] Lu, (**e**) [^177^Lu]Lu-FAPI-04, (**f**) [^177^Lu]Lu-FAPI-46. TLC plate: ITLC SG, mobile phase: 0.05 M Citrate buffer pH 4, Free [^177^Lu] Lu RF: 0.8–1.0, [^177^Lu]Lu-FAPI-04 & [^177^Lu]Lu-FAPI-46. RF: 0.0–0.2

### Stability experiments of [^177^Lu]Lu-FAPI radiopharmaceuticals

#### Stability of [^177^Lu]Lu-FAPI-04 & [^177^Lu]Lu-FAPI-46 in final formulations

**Fig. 6:**
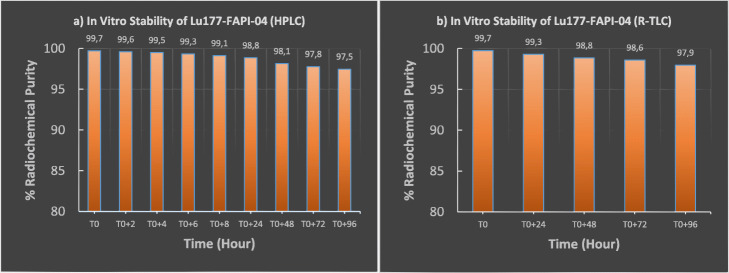
Stability study of [^177^Lu]Lu-FAPI-04. **a** Radiochemical purity results analyzed by R-HPLC: T0, T0 + 2 h, T0 + 4 h, T0 + 6 h, T0 + 8 h, T0 + 24 h, T0 + 48 h, T0 + 72 h, T0 + 96 h. **b** Radiochemical purity results analyzed by R-TLC: T0, T0 + 24 h, T0 + 48 h, T0 + 72 h, T0 + 96 h**.** *(T0 = End of the synthesis)*

**Fig. 7:**
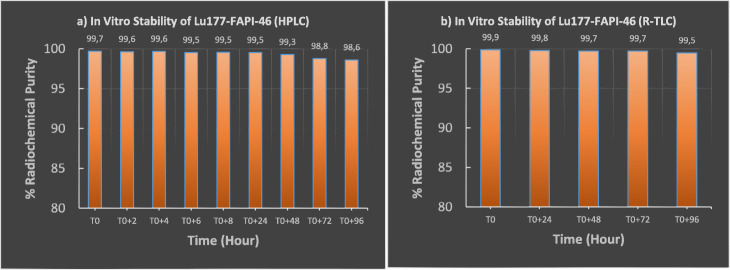
Stability study of [^177^Lu]Lu-FAPI-46. **a** Radiochemical purity results analyzed by R-HPLC: T0, T0 + 2 h, T0 + 4 h, T0 + 6 h, T0 + 8 h, T0 + 24 h, T0 + 48 h, T0 + 72 h, T0 + 96 h.**, b** Radiochemical purity results analyzed by R-TLC: T0, T0 + 24 h, T0 + 48 h, T0 + 72 h, T0 + 96 h**.** *(T0 = End of the synthesis)*

The stability of [^177^Lu]Lu-FAPI derivatives were monitored for 4 days from the end of synthesis **(T0)** in room conditions 24 °C (Figs. 6 and 7). Radiochemical purity analyzes were performed by R-HPLC and R-TLC using the same methods indicated in Figs. 4 and 5.

#### Stability of [^177^Lu]Lu-FAPI-04 & [^177^Lu]Lu-FAPI-46 in presence of human serum

**Fig. 8:**
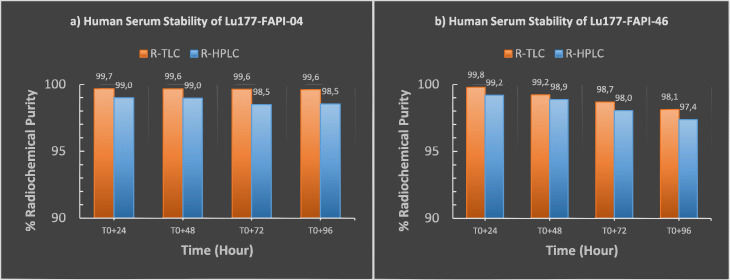
Human serum stability study of [^177^Lu]Lu-FAPI-04 & [^177^Lu]Lu-FAPI-46 at 37 °C ambient temperature. **a** Radiochemical purity results of [^177^Lu]Lu-FAPI-04 analyzed by R-TLC & R-HPLC: T0 + 24 h, T0 + 48 h, T0 + 72 h, T0 + 96 h. **b** Radiochemical purity results of [^177^Lu]Lu-FAPI-46 analyzed by R-TLC & R-HPLC: T0 + 24 h, T0 + 48 h, T0 + 72 h, T0 + 96 h**.** *(T0 = End of the synthesis)*

Mixtures of 1 mCi final product / 1 mL human serum were prepared and incubated at 37 °C degrees. The stability of the mixtures was monitored for 4 days (Fig. 8). Radiochemical purity analyzes were performed by R-HPLC and R-TLC using the same methods indicated in Figs 4 and 5.

## Results and discussion

A crucial case for radionuclide-based clinical administration is the synthesis procedure manually or through an automated system. The majority of the therapeutic radiopharmaceuticals are still prepared manually although this process fundamentally causes radiation exposure and risk of contamination (Meyer et al. 2004). An automatically synthesis of radiopharmaceuticals donates standardization, safety dose, stability, reproducibility and high yield (Velikyan 2015). Moreover, this process provides a GMP-compliance production in clinical studies and disposable cassette systems are utilized to prevent cross-contamination coming from tubing systems, which leads to an exact sterility and high purity. (Boschi et al. 2013).

ML Eazy synthesis device is a fully user-defined system combined by valves, sensors, pump and other equipment. This practical design provides a flexibility option for the preparation of various radiopharmaceuticals and it is frequently utilized for [^68^Ga] Ga and [^177^Lu] Lu based radiosynthesis (Persico et al. 2020). More recently, Spreckelmeyer et al. has successfully described the synthesis of [^68^Ga]Ga-FAPI-46 on a ML Eazy synthesis module (Spreckelmeyer et al. 2020). Considerable attention has been devoted to theranostic studies in nuclear medicine, therefore we have developed a fully automated synthesis method for [^177^Lu]Lu-FAPI-04 and [^177^Lu]Lu-FAPI-46 on the same module (ML Eazy, Fig. 3). Thus, further multi-center pre-clinical and clinical trials with FAPI based radiopharmaceuticals can be easily performed for theranostic purposes in the same commercially available synthesizer.

In our experiments, the amount of precursor and pH medium were kept constant due to the previously optimized parameters for well-known [^177^Lu]Lu-PSMA and DOTATATE synthesis. Table 4 summarizes the results after radiolabeling process. The radiochemical yield was around 85–90% with absolute radiochemical purity (99%). Another important parameter is amount of radionuclide (mCi) about administration dose for patients. More recently, Altmann et al. has published a comprehensive review related to novel clinical trials about FAPI based radiopharmaceuticals (Altmann et al. 2020; Ballal et al. 2020). In this article, clinical studies indicated [^90^Y][Y] and [^177^Lu][Lu] FAPI derivatives were successfully applied to patients with relatively low dose of 70 mCi without any side effect. Therefore, amount of activity was nearly scaled up to 100 mCi to check any dramatic change about RCY and RCP. Experimental results showed there was no significant difference with increase amount of radionuclide activity. R-HPLC and R-TLC analyses indicated there was a trace amount of free and colloidal [^177^Lu] Lu after completion of the reaction (Fig. 5). Citrate buffer mobile phase was exclusively afforded as a mobile phase, and different RF values were well recorded on TLC analysis. All reactions were tried as three times for validation of radiochemical yield and radiochemical purity.

**Table 4:** The final product specifications for [^177^Lu]Lu-FAPI-04 and [^177^Lu]Lu-FAPI-46 *(n = 3, 100 mCi, [*^*177*^*Lu] Lu & 100* μg *of FAPI derivatives)*

Test	[^**177**^Lu]Lu-FAPI-04 (***n*** = 3)	[^**177**^Lu]Lu-FAPI-46 (***n*** = 3)
Radiochemical yield	88% ± 3	86% ± 3
Radiochemical purity (R-HPLC)	≥ %99	≥ %99
Radiochemical purity (R-TLC)	≥ %99	≥ %99
pH	4,5–8	4,5–8
Appearance	Clear, Colorless	Light yellow
Volume	15–20 mL.	15–20 mL.
Radioactivity concentration	4–6 mCi/ml	4–6 mCi/ml

As known that, specific uptake, biodistribution, and longer tumor retention time are vital requirements for an administration of [^177^Lu] Lu, which is well known therapeutically effective longer-lived radionuclide. For this reason, within the scope of stability studies, radiochemical purity analyzes were comprehensively investigated by R-TLC and R-HPLC for up to 4 days (Figs. 6, 7 and 8). Stability studies were divided into two parts; in laboratory medium at 24 °C and in human serum at 37 °C. First, FAPI-04 and FAPI-46 based compounds were respectively submitted to stability experiments at room temperature. Radiochemical purity results indicated those compounds are highly stable at room temperature up to 4 days confirmed by both R-TLC and R-HPLC analysis (Figs. 6 and 7). Similar results were also observed regarding serum stability (Fig. 8).

## Conclusion

In conclusion, a fully automated synthesis of [^177^Lu] Lu labeled FAPI derivatives have been remarkably presented for the first time. The evaluation of experimental records revealed that the automated synthesis provided a complete radiolabeling process with high yield, high reproducibility and more than 99% radiochemical purity. All synthesis steps were implemented in the synthesis template without any manual interaction. Disposable cassette was employed to prevent cross-contamination and radiation exposure. Detailed QC and stability studies were well presented and all final product specifications were obtained within limits and acceptable criteria. Our work could lead to a practical theranostic application for harmonized and standardized multicentre clinical trials.
