# Correction to: Fully-automated synthesis of 177Lu labelled FAPI derivatives on the module modular lab-Eazy

**DOI:** 10.1186/s41181-021-00134-z

**Published:** 2021-05-19

**Authors:** Kurtulus Eryilmaz, Benan Kilbas

**Affiliations:** Moltek A. S. Gebze Organize Sanayi, 41400 Gebze, Kocaeli Turkey

**Correction to: EJNMMI Radiopharm Chem 6, 16 (2021)**

**https://doi.org/10.1186/s41181-021-00130-3**

After publication of the original article (Eryilmaz and Kilbas 2021), the authors identified an error in Figs. 1 and 2. The correct figures are given below.

**Fig. 1:**
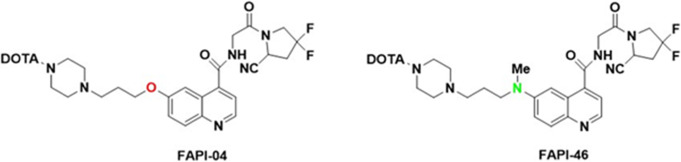
Molecular Structures of FAPI-04 and FAPI-46

**Fig. 2:**
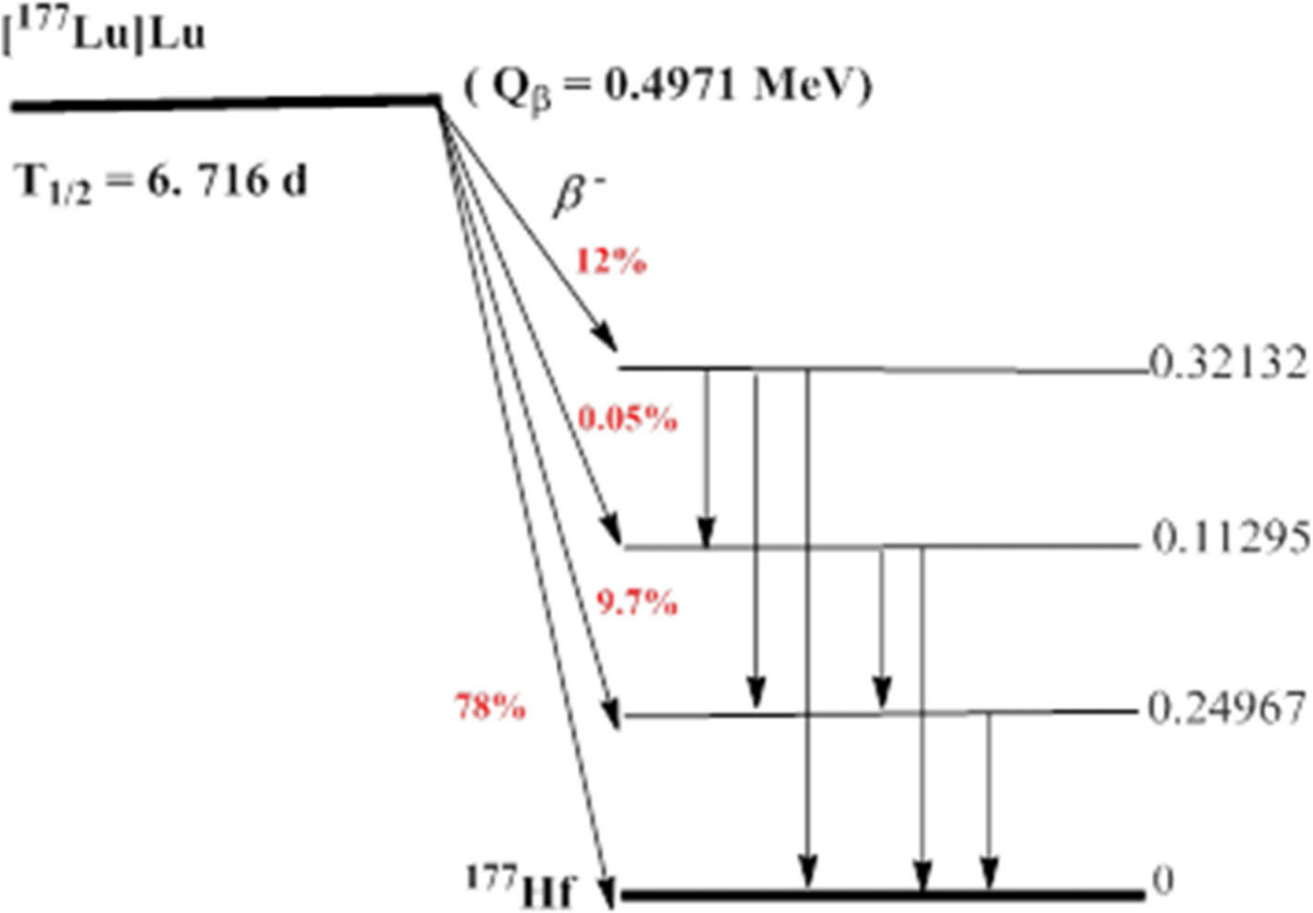
Decay chain scheme of [177Lu]Lutetium

The original article has been corrected.
